# Optimal Period of Calcium Propionate Supplementation in Arrival High-Risk Bull Calves: Growth Performance, Body Fat Reserves, and Serum Metabolites

**DOI:** 10.3390/ani15081170

**Published:** 2025-04-18

**Authors:** Daniel Rodríguez-Cordero, Octavio Carrillo-Muro, Pedro Hernández-Briano, Paola Isaira Correa-Aguado, Alejandro Rivera-Villegas, Alberto Barreras, Rosalba Lazalde-Cruz, Richard A. Zinn, Alejandro Plascencia

**Affiliations:** 1 Unidad Académica de Medicina Veterinaria y Zootecnia, Universidad Autónoma de Zacatecas, General Enrique Estrada 98500, Mexico; danielroco@uaz.edu.mx (D.R.-C.); pedro.hernandez@uaz.edu.mx (P.H.-B.); paocorrea@uaz.edu.mx (P.I.C.-A.); alejandro.rivera@uaz.edu.mx (A.R.-V.); 2 Instituto de Investigaciones en Ciencias Veterinarias, Universidad Autónoma de Baja California, Mexicali 21100, Mexico; beto_barreras@yahoo.com (A.B.); rosalba.lazalde@uabc.edu.mx (R.L.-C.); 3 Animal Science Department, University of California, Davis, CA 95616, USA; razinn@ucdavis.edu; 4 Facultad de Medicina Veterinaria y Zootecnia, Universidad Autónoma de Sinaloa, Culiacán 80260, Mexico

**Keywords:** costs, energy diet, gluconeogenic precursors, income, newly received calves

## Abstract

Fifty high-risk bull calves were used in a completely randomized design (ten calves/treatment) to evaluate the optimal period of calcium propionate (CaPr) supplementation following arrival into the feedlot. The variables evaluated were the growth performance, dietary energetics, body fat reserves, serum metabolites, and economic return at day 56 of arrival. Calves, which were weighed at the moment of reception (156.2 ± 1.57 kg off-truck body weight, BW), were received with a 50:50 forage-to-concentrate ratio diet and clean water. At 12 h from reception, they were housed in individual pens during a 56 d feeding period and assigned to treatments consisting of a daily supplementation of a commercial product (Propical^®^ Dresen Química, SAPI de CV., Mexico City, Mexico) that provided 19 g CaPr/calf for 0, 14, 28, 42, or 56 d following arrival into the feedlot. Compared with the other treatments, the calves receiving CaPr during the initial 42 d had a greater average daily weight gain (*p* ≤ 0.035) without affecting the dry matter intake (*p* ≥ 0.24). Thus, the gain efficiency (*p* ≤ 0.050) and dietary energy utilization were improved (*p* ≤ 0.046). Rib fat thickness (*p* ≥ 0.090) and *Longissimus lumborum* muscle area (*p* ≥ 0.112) were not affected by the CaPr supplementation, whereas calves showed the greatest values (*p* ≤ 0.038) to the rump back fat thickness at 42 and 56 days of CaPr supplementation. With the exception of the total albumin (being maximal at day 56 (*p* ≤ 0.024)) and total cholesterol (which, compared to the controls, was maximal at 28 and 42 d; *p* = 0.030), the serum metabolic profiles were not affected by the treatments. Using the profit estimated for the control group as a baseline, supplementing CaPr for 42 d yielded a greater net income (USD 34.84 more/calf). CaPr supplementation for more or less than 42 d showed a very similar profit (~USD 6.80/calf). Because the cost of gain was very similar for these treatments and the controls (USD 1.42 vs. 1.46/kg), the positive difference in profit was mediated mainly by an increase in income selling (+USD 13.02/calf) for the CaPr calves. Based on the performance, serum metabolites, and profit, it is concluded that the optimal duration of supplemental CaPr is 42 d. Offering 19 g CaPr during this period enhanced the growth performance, dietary energy, and economic returns.

## Simple Summary

Much of the cattle received in feedlots are considered “high risk”. During the first week following arrival, the feed intake is low. This, in combination with transportation and marketing, shrinks the result to a negative energy balance, reducing body fat reserves and depressing immune function. Calcium propionate (CaPr), a gluconeogenic precursor, has shown positive results when supplemented at a rate of 20 g daily to calves during the first 8 weeks following arrival in the feedlot. The currently recommended period for CaPr supplementation is the initial 56 d following arrival. However, the optimal period of supplementation has not been evaluated. Therefore, different periods of CaPr supplementation were evaluated (14, 28, 42, or 56 d). Responses in terms of the growth performance, dietary energetics, body fat reserves, and economic returns were optimal when high-risk calves received CaPr during the initial 42 d following arrival into the feedlot. Therefore, supplementing CaPr beyond 42 d did not represent a growth performance, feed efficiency, health, nor economic return advantage.

## 1. Introduction

Twenty-eight percent of cattle placed in feedlots in North America are considered “high risk” [1]. This effect is attributable to (a) an unknown health and management background; (b) a light-at-arrival body weight (less than 200 kg BW); (c) an age of less than 7 months; (d) being weaned for a maximum of 14 days; (e) rough handling and/or long transportation; (f) being commingled with calves from different sources; (g) being unvaccinated prior to arrival at the feedlot [2].

The primary challenge for high-risk received calves at the feedlot during the first 14 d upon arrival is depressed (less than 1.5% of BW) dry matter intake (DMI) [3,4,5,6]. A low DMI promotes a negative energy balance, thus reducing body fat reserves and negatively affecting the immune function, and increasing the risk of the presentation of respiratory diseases [7,8].

Research evaluating gluconeogenic precursors for the enhancement of the rate of recovery of the energy balance in stressed calves has gained increasing interest [9,10,11,12]. In this sense, calcium propionate (CaPr) has been shown to have a positive effect on ruminal bacterial diversity, thereby improving ruminal fermentation (increasing the propionate/acetate ratio) and reducing N losses. In addition, CaPr improves the glucose metabolism and adipogenesis. All of these effects impact positively on the body energy status, mainly in cattle that show depressed feed intake or in stressed cattle [9,10].

Although information regarding the effect of supplemental CaPr in newly received calves in the feedlot is very limited, it has been shown that the inclusion of a daily dose of 20 g CaPr/calf in the feed during the initial 56 d enhanced the average daily gain (ADG) and body fat reserves [13]. Furthermore, it has been determined that the concentration level affects the potential response to CaPr in newly received calves; in this way, the growth performance response to supplemental CaPr was greater for calves fed a receiving diet containing 50% concentrate [14]. Therefore, it can be assumed that the appropriate dose and the type of diet to obtain the greatest benefits from CaPr supplementation in received cattle are known. However, the optimal period of supplementation has not yet been evaluated.

After arriving into the feedlot, clinically healthy calves recover lost body weight and water, recover their immune capacity, and establish a social structure within a few days. With time, the calves gradually adapt to their new surroundings, while also improving their energy consumption and health. For this reason, it is possible that the optimal benefits of CaPr supplementation will only be visible for a limited time. Hence, the objective of this study was to determine the optimal period of calcium propionate supplementation (19 g/calf/d) in high-risk arrival calves feeding on a 50% concentrate diet during the first 56 d from arrival on the growth performance, body fat reserves, and serum metabolites. In addition, an analysis of the cost–profit strategy of supplementation was performed.

## 2. Materials and Methods

### 2.1. Animal Care and Use

This experiment was conducted at the Torunos Livestock Preconditioning Center, the Grupo Exportador Pa Lante S.P.R. de R.L., in Fresnillo, Zacatecas, Mexico (23°08′56.22′′ N and 102°43′48.57′′ W) from April to June 2024. All of the experimental procedures used in this study were approved by the Official Mexican norms for animal care [15,16], and the experimental protocols were reviewed and approved by the Unidad Académica de Medicina Veterinaria y Zootecnia at the Universidad Autónoma de Zacatecas (UAMVZ-UAZ)—Institutional Bioethics and Animal Welfare Committee (Protocol # 2024/04/08).

### 2.2. Experimental Animals

All of the calves used in this experiment were within the definition of “high risk” described by Carrillo-Muro et al. [17]. Briefly, the calves are classified as high risk due to being recently weaned, having received no vaccinations, not having been castrated or dehorned, being comingled, and having been moved through an auction market. One hundred and six recently weaned Continental × British crossbred bull calves from different locations within Zacatecas State were purchased and transported (approximately 120 km, equivalent to 4 h by truck) to the Torunos Livestock Preconditioning Center on 12 April 2024. Upon arrival at the Torunos Livestock Preconditioning Center, all of the bull calves were provided oats hay and unlimited access to water during the first 12-h from arrival. On the following morning (0600 h), 13 April 2024, the bull calves were weighed, vaccinated (Biovac 11 Vías^®^, Biozoo, Zapopan, Jalisco, Mexico), treated against parasites (4% ivermectin; Master LP^®^ injectable, Ourofino Salud Animal, São Paulo, Brazil), and received a metaphylactic treatment with oxytetracycline (5 mg/kg BW; liquid Emycin^®^, Zoetis, Ciudad de Mexico, Mexico). Immediately after handling, from the original group, 50 calves (156.2 ± 1.57 kg BW, 6–7 months of age, and evaluated as “high risk”) were selected for their inclusion in the study and individually allotted to 50 individual pens (3.14 m × 5.25 m; 10 pens/treatment) with a soil (leptosol type) pen floor, and individual feed bunks and water drinkers; then, the calves received a 50:50 forage-to-concentrate ratio diet through the experimental period. The calves were evaluated daily by a qualified a veterinarian for any signs of bovine respiratory distress, including labored breathing, nasal or ocular discharge, depression, anorexia, lethargy, or lack of appetite. No morbidity or mortality was observed during the study.

### 2.3. Treatments and Diets

**Figure 1:**
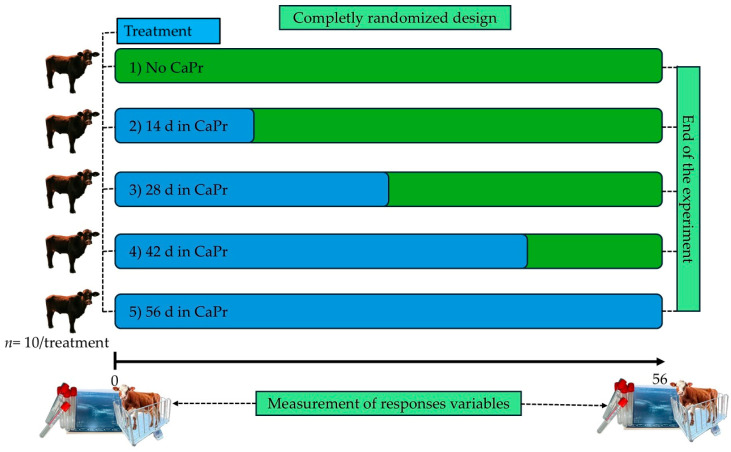
A completely randomized design experiment using 50 individually housed calves (10 calves/treatment). The treatments consisted of the oral administration of calcium propionate (CaPr) at a dose of 19 g/calf/d at five supplementation periods of 0, 14, 28, 42, or 56 d following arrival. The non-supplemented group (fully green) was used as a control group.

Since the net ingestion of 13.5 g of propionate has been shown to be optimal in newly receiving calves (equivalent to 19 to 20 g of CaPr) [13,14], the calves were randomly assigned to treatments consisting of the oral administration of CaPr at a dose of 19 g/calf/d at the following five supplementation periods: 0, 14, 28, 42, or 56 d after the calves arrived at the feedlot (Figure 1). The source of the CaPr used was Propical^®^ (Dresen Química, SAPI de CV., Mexico City, Mexico), which contains 73% of propionic acid (the net daily ingestion of propionic acid by calves receiving CaPr was 13.87 g).

The basal diet (Table 1) was formulated to contain 15% crude protein and provided 0.98 Mcal/net energy for gain (NE_g_); the diet included 50% roughage (alfalfa hay mature and oats hay) and 50% of concentrates (cracked corn grain, soybean meal, soybean oil, and molasses, and vitamin and mineral premix) to meet the requirements for growing bull calves [18].

To ensure that the total dosage of CaPr was ingested, half of the dose was mixed with 100 g of the basal diet and provided twice daily at 0800 and 1600 h before the basal diet was offered. Fresh feed was provided three times a day, at 0800, 1200, and 1600 h. Every morning, approximately 30 min before the morning feed, the feed bunks were checked to assess the previous day’s intake. Any feed refusal was removed, weighed, and recorded. This information was then used to adjust the 1600 h feed so to ensure a refusal rate of less than ~100 g/calf; the amount of feed given at 0800 and 1200 h remained constant.

Feed and feed refusal samples were collected daily for the following analyses: DM (oven drying at 105 °C until no further weight loss; method 930.15) and CP (N × 6.25; method 984.13) according to the AOAC [19]. Neutral detergent fiber (NDF) was determined following the procedures described by Van Soest et al. (corrected for NDF-ash, incorporating heat-stable amylase using Ankom Technology, Macedon, NY, USA) [20]. All of the analyses were performed in the Animal Nutrition Laboratory from the UAMVZ-UAZ.

### 2.4. Evaluation of Productive Performance

To evaluate the impact of the treatments on the growth performance, the calves were weighed at the start and end of the experiment (56 d); the initial weight was off-truck (initial shrink weight, ISBW), while the final weight was multiplied by 0.96 (pencil shrink) to account for gastrointestinal fill (final shrunk body weight, FSBW). The calculations of the ADG, dry matter intake (DMI), and feed efficiency (ADG/DMI ratio) were estimated as follows: (1) ADG = [(Weight out − Weight in/56 d] expressed as kg/d; (2) DMI = (Feed offered − Feed refused), which was weighed and recorded daily, expressed as kg/d; (3) ADG/DMI ratio = (ADG/DMI).

The net energy (NE) for maintenance (NE_m_) was calculated using the following quadratic equation described by Zinn et al. [21]:

$$x=\frac{-b\pm \sqrt{b^{2}-4ac}}{2c}$$

where *a* = − 0.41 × EM; *b* = (0.877 × EM) + (0.41 × DMI) + EG; *c* = − 0.877 × EM, where EM = the requirement of energy for maintenance and EG = the requirement of energy for gain.

The requirement of energy for maintenance (EM, Mcal/d) was estimated using EM = 0.077 × SBW^0.75^, where the average SBW = ISBW + FSBW/2 [22] and the requirement of energy for gain (EG, Mcal/d) was estimated using the equation 0.0557 × SBW^0.75^ × ADG^1.097^ [18,21]. The performance-calculated NE_g_ was subsequently derived from NE_m_ (NE_g_ = 0.877 × NE_m_ − 0.41), as previously described by Zinn et al. [21]. The estimation of the expected DMI was performed based on the observed ADG, the average SBW, and the estimated NE values of the diet (Table 1), as follows: expected DMI, kg/d = (EM/NE_m_) + (EG/NE_g_), where NE_m_ and NE_g_ are the tabular NE values of the diet based upon the formulation [18]. The efficiency of the dietary energy utilization in the growth performance trials was evaluated by using the ratio of the observed-to-expected DMI and observed-to-expected dietary NE.

### 2.5. Body Fat Reserves and Longissimus lumborum Muscle Area

The body fat reserves and *Longissimus lumborum* muscle area were obtained through ultrasonography at days 0 and 56 by a certified technician using a real-time scanner equipped with a linear array transducer at 3.5 MHz (Aloka Prosound 2 instrument, TP Global Medical Equipment, Querétaro, México). Before capturing the images, the area to be imaged was clipped using hair clippers, cleaned using compressed air, and ultrasound gel was applied as the couplant. Measurements of the rib fat thickness (FAT, mm) and *Longissimus lumborum* muscle area (LMA, cm^2^) were taken between the 12th and 13th ribs. The rump back fat thickness (RFT, mm) was taken at the P8 site (at the intersection of a line going forward from the pin and a line down from the high point in the hindquarter).

### 2.6. Assessment of Enzymimatic Activity and Serum Metabolites

All of the blood samples to determine serum metabolites were processed in the Veterinary Clinical Analysis Laboratory from the UAMVZ-UAZ.

Blood samples (10 mL) were obtained from all calves on days 0 and 56. The calves were restrained in a standing position in a squeeze chute (Priefert^®^, Model S0191, Mount Pleasant, TX, USA). After preparing the venipuncture site with a gauze swab soaked in 70% alcohol, the blood was collected before morning feeding (approximately 0700 h) by jugular venipuncture using an 18-gauge × 3.81 cm needle.

Blood samples for serum were collected in a 5.0-mL BD Vacutainer SST and centrifuged (2500× *g* at 4 °C for 15 min) within 15 min of collection. After collection, the samples were stored in coolers (4 °C) and transported directly to the university laboratory, approximately 40 km away. Upon arrival, the samples were analyzed within 2 h of collection.

The enzymatic activity and serum metabolites were quantified with an automated analyzer (FUJI DRI-CHEM NX500^®^; Fujifilm, Tokyo, Japan), using the proper kits for each metabolite from the same enterprise (Fujifilm, Tokyo, Japan). The following parameters were determined: the activity of alkaline phosphatase (ALP), gamma glutamyltransferase (GGT), aspartate aminotransferase (AST), and alanine aminotransferase (ALT); the levels of the total protein (TP), albumin (ALB), globulin (GLO = TP − ALB), blood urea nitrogen (BUN), creatinine (CRE), total bilirubin (TBIL), total cholesterol (TCHO), triglyceride (TG), calcium (Ca), glucose (GLU), sodium (Na^+^), potassium (K^+^), and chlorine (Cl^−^). The globulin fraction (GLO) was a calculated value obtained by subtracting the ALB concentration from the TP concentration.

### 2.7. Cost and Income Analysis

Economic analysis and cost of gain were performed with growth performance (SIBW, SFBW, and DMI) data by using the US dollar as the currency (USD).

Calculations were performed as follows: (1) processing practice = metaphylactic antimicrobial treatment + vaccination + deworming + pour-on cypermethrin + ear tag; (2) feed cost = (DMI, kg/d × price of feed kg) × days on feed; (3) CaPr supplementation = (CaPr, kg/d × price of CaPr kg) × days on supplementation; (4) cost total = processing practice + feed + CaPr supplementation; (5) income (selling calves) = (weight out − weight in) × price of BW/kg to calves; (6) net income = income (selling calves) − total cost; (7) difference = CaPr treatments–control; (8) cost of gain = total cost/(SFBW − SIBW).

The analysis considered data for a 56 d period, calculating the costs (processing practice, feed, and CaPr supplementation), incomes (net income and difference), and cost of gain.

The price of feed (USD 0.349/kg) and CaPr (USD 2.1/kg), metaphylactic antimicrobial treatment (USD 1.38/calf), vaccination (USD 0.43/calf), deworming (USD 0.75/calf), pour-on cypermethrin (USD 0.63/calf), and ear tags (USD 0.61/calf) were obtained from FORRVET S.A. de C.V (Forrajera y Veterinaria, Durango, México). The price of calves (USD 3.62/kg BW) was obtained from the PaLante enterprise for the Zacatecas region.

The program Excel^®^ (Office 365, Microsoft, Redmond, WA, USA) was used for the cost and income calculations. To compare the cost of supplementation, the profit estimated for the control group was used as a baseline and the results were compared between treatments using descriptive statistics.

### 2.8. Statistical Analyses

The following data were analyzed as a completely randomized design with 10 replicates/treatment under following additive model:

*Y*ij = μ + τi + εij,

where Yij is the response variable, μ is the common experimental effect, τj is the treatment effect, and εijk is the residual error.

The following variables were analyzed: (1) the growth performance (ADG, DMI, and the ADG/DMI ratio); (2) the dietary energy, and the efficiency of dietary NE utilization and retention; (3) the body fat reserves (FAT and RFT) and LMA; (4) the enzymic activity and metabolites, all using the PROC GLM procedures of SAS^®^ software 9.3 [23]. Individual calves served as the experimental unit.

The data of the SIBW, body fat reserves, LMA, enzymic activity, and metabolites, taken at day 0, were used as covariates to the measurements obtained on day 56.

Tukey’s multiple comparison procedures were used, and the treatment effects were considered significant when the *p*-value was ≤0.05. Comparisons between the differences in economic income/cost from the calves without CaPr and supplemented with CaPr were performed with the *t*-test using PROC TTEST in SAS^®^ software 9.3; Cary, NC, USA [23].

## 3. Results

### 3.1. Growth Performance, Dietary Energetics, Body Fat Reserves, and Longissimus Muscle Area

Based on the observed DMI, the dose of 20 g/calf/d resulted in an average of 4 g CaPr concentration/kg diet. The net daily propionate ingestion was 13.9 g/calf. This propionate net ingestion is similar to the 13.8 g/d of propionate ingested in previous studies, in which the propionate supplementation showed benefits in the growth performance and health in arrival calves [13,14].

There were no treatment effects (*p* ≥ 0.24) on the DMI, which averaged 4.93 ± 0.11 kg. Compared to the rest of the treatments, the calves that received CaPr for 42 d had a greater (*p* ≤ 0.034) average daily weight gain. Accordingly, supplementing CaPr for 42 d enhanced both the gain efficiency (15.5%; *p* = 0.008) and the dietary NE (9.6%; *p* = 0.004) when compared to the controls; when compared with the calves that received CaPr supplementation for 14, 28, and 56 d, the 42 d supplementation enhanced the gain efficiency (10.8%; *p* ≤ 0.050) and dietary NE (5.7%; *p* ≤ 0.046) (see Table 2 and Table 3).

Even though CaPr supplementation for 14, 28, and 56 d numerically increased the ADG (5.3%; *p* ≥ 0.321) and dietary energetic efficiency (11.6%; *p* ≥ 0.143), these increases were not statistically significant. In the same manner, the FAT (*p* ≥ 0.09) and LMA (*p* ≥ 0.112) were not affected by the CaPr supplementation, whereas the calves showed the greatest values (*p* ≤ 0.038) to the RFT at 42 and 56 d of CaPr supplementation.

### 3.2. Enzymatic Activity and Serum Metabolites

Treatment effects on the enzymic activity and serum metabolites are shown in Table 4, Table 5 and Table 6. With the exception of the total albumin (being maximal at day 56 (*p* ≤ 0.024)) and total cholesterol (which, compared to the controls, were maximal at 28 and 42 d; *p* = 0.030), the blood enzymatic activity and metabolic profiles were not appreciably affected by the treatments.

### 3.3. Cost and Income Economics Analysis

The cost analysis results are shown in Table 7. Using the profit estimated for the control group as a baseline, the bull calves that received CaPr supplementation for 14, 28, and 56 d showed a very similar profit (~6.80 USD/calf). Because the cost of gain was very similar for these treatments and controls (USD 1.42 vs. 1.46/kg), the positive difference in profit was mediated mainly by an increase in income selling (+USD 13.02/calf) for the CaPr calves. For the calves that received CaPr for 42 d, compared to the controls, the profit controls were greater by +USD 34.84/calf, both due to an increase in income selling (+USD 41.99/calf) and a reduction in the cost of gain (USD 1.26 vs. 1.46/kg BW).

## 4. Discussion

To date, the ideal dietary energy density of newly received calves in the feedlot remains uncertain. Increasing the dietary energy density by increasing the starch concentration can improve the daily weight gain but may also increase the risk of health problems [24,25,26]. Attempts have been made to increase the caloric availability without negatively affecting cattle health through various feeding strategies, such as fat supplementation [27,28,29]. As an alternative to starch in ruminant diets, research regarding the gluconeogenic precursors glycerol and CaPr have gained increasing interest in dairy [30] and feedlot cattle [9]. Although information regarding the effect of supplemental CaPr in newly received calves in the feedlot is very limited, it has been shown that the inclusion of a daily dose of 20 g CaPr/calf during the initial 56 d on feed enhanced the ADG and body fat reserves [13]. Furthermore, it has been determined that the concentration level affects the potential response to CaPr in newly received calves; in this way, the growth performance response to supplemental CaPr was greater for the calves fed a receiving diet containing 50% concentrate [14]. Therefore, it can be assumed that the appropriate dose and type of diet to obtain the greatest benefits from CaPr supplementation in received cattle are known. However, the optimal period of supplementation has not yet been evaluated.

After arriving into the feedlot, clinically healthy calves recover lost body weight and water, recover immune capacity, and establish a social structure within a few days. With time, the calves gradually adapt to their new surroundings, thereby improving their energy consumption and health. For this reason, it is possible that the optimal benefits of CaPr supplementation will only be visible for a limited time. Hence, we hypothesized that CaPr supplementation (19 g/calf/d) has an optimal period in high-risk arrival calves feeding a 50% concentrate diet during the first 56 d from arrival to the feedlot. Due to the natural adaptation of cattle, this period could be less than 56 d (considered the bottom time from the arrival period).

### 4.1. Energetics

Multiple effects have been observed in response to CaPr consumption. At the ruminal level, it promoted changes in the population and the diversity of ruminal microorganisms, which alter fermentation patterns by increasing the proportion of propionate and decreasing CH_4_ production [31,32,33]. It is well known that roughly 2–12% of gross energy intake is lost as methane. Methane production is highly positively associated with the dry matter intake, live weight, and average daily gain. In addition, methane production also has low-to-moderate positive associations with carcass composition traits, such as the rib fat, rump fat, intramuscular fat, and ribeye area. Therefore, a slight reduction in enteric methane production could reflect a better performance and efficiency in cattle.

Additionally, the presence of CaPr favored the greater ruminal fermentation of DM, reduced the protein degradation, and reduced the NH_3_-N and BUN [13,34,35]. Furthermore, CaPr also improved the insulin response in the glucose metabolism [36,37,38] and increased adipogenesis [39]. A complex relationship exists between the nutrient intake and growth performance potential. Approximately 80% of the total tract digestible organic matter intake is fermented in the rumen, so optimizing rumen fermentation for nutrients is key to improving dietary energy utilization. The benefits of improvements on DM digestibility could become more pronounced when cattle show a depressed DMI, as is the case for newly received calves at the feedlot. Together, all of these mechanisms may promote an improved energy status and nutrient retention, resulting in a better growth rate and feed efficiency.

By comparing the observed dietary net energy with the expected dietary net energy based on the growth performance, growing–finishing trials can determine how efficiently energy is utilized; this derivation is a more precise tool than the conventional measure of “feed efficiency” [21]. The estimation of the dietary NE based on the growth performance provides valuable information about the potential effects of treatment (or environment) on the efficiency of dietary energy utilization. An observed-to-expected dietary NE ratio of 1.00 indicates that the ADG is consistent with the formulated dietary NE values based on the tables of feedstuff standards and the observed DMI [18]. A ratio that is greater than 1.00 indicates a greater efficiency of dietary energy utilization, whereas a ratio that is lower than 1.00 indicates a lower-than-expected efficiency of energy utilization. Based on the above, the control calves showed a 20% greater efficiency from the expected. This can be explained partially by compensatory growth and by rehydration during the first days from arrival. Even so, the calves that received CaPr for 42 d showed a 32% greater efficiency from the expected (9.6% greater energy efficiency than the controls); the difference between the controls and the supplemented calves is a reflection of the better utilization of the feed energy in the supplemented calves. The greater rump fat thickness value for the calves on the 42 d supplementation reinforces this energy utilization improvement.

### 4.2. Growth Performance, Body Fat Reserves, and Longissimus Muscle Area

In the current study, CaPr did not affect the DMI. CaPr consumption at concentrations over 12 moles per day had a hypophagic effect in dairy cattle [40,41]. This quantity of CaPr is 6-fold greater than the CaPr concentration/kg diet used in the current study.

It has been previously reported that CaPr supplementation enhanced the ADG, improved the feed efficiency, and favored changes in the body fat deposition in finishing lambs. In this sense, Carrillo-Muro et al. [10] observed that daily CaPr supplementation (10 g/lamb) for 42 d increased the DMI (13%), ADG (28%), and ADG/DMI ratio (17%). Likewise, Martinez-Aispuro et al. [42] observed an increased ADG and ADG/DMI ratio in finishing lambs receiving a daily dose of 13.9 g CaPr. Cifuentes-López et al. [43] observed positive effects on the carcass parameters (LMA and FT), but the supplementation did not affect the growth performance. In other studies [44,45], CaPr supplementation affected neither the growth performance nor the carcass parameters in lambs. In a meta-analysis [9], CaPr supplementation in finishing lambs did not affect the DMI or carcass yield, but enhanced the ADG and gain efficiency.

Information on the benefits of the CaPr supplementation of feedlot cattle is limited. However, Rodríguez-Cordero et al. [13] observed that a daily dose of 20 g CaPr/calf promoted increased ADG, gain efficiency, and body fat reserves in newly received calves during the initial 56 d following arrival into the feedlot. Rivera-Villegas et al. [14] noted that the response to CaPr supplementation of receiving cattle was more appreciable in calves fed less energy dense diets (50% concentrates).

Since there were no differences in the metabolites involved in energy processes or in growth, the reason why calves improved in their daily gain up to 42 d, and then showed a slight decline when reaching 56 d, is uncertain and requires further research. However, it is apparently related to the natural adaptation of cattle to new surroundings. Within a few days following arrival into the feedlot, clinically healthy calves regain lost water and body weight, recover their immune capacity, and establish a social structure. In a natural way, calves gradually adapt with time following arrival, improving their energy consumption and health status. Thus, the expected optimal benefits of CaPr supplementation (and other strategies that improve the energy availability) may be apparent for a limited time period.

The improved growth performance observed in the present study is consistent with previous studies [13,14] in which supplementation with 20 g of CaPr during the initial 56 d enhanced the growth performance. However, based on the growth rate, the efficiency of dietary energy utilization, and fat deposition, the greatest benefit occurred when the calves received CaPr supplementation for the first 42 d. This result could be an indicator that calves reach more adequate physiological and metabolic conditions when they surpass 42 d from arrival, since a better response to CaPr supplementation is expected when metabolic conditions and energy consumption are compromised. This result is in line with the generalization that the more significant improvements in growth performance and health are observed during the first 30–40 d from arrival [46,47,48].

### 4.3. Enzymatic Activity and Serum Metabolites

Consistent with previous studies [13,14], the calves in all of the treatments maintained the metabolites studied within the normal reference intervals (RIs) for receiving calves in similar conditions [17]. The serum metabolites evaluated in the current study are indicators of renal and hepatic function, tissue damage, bone growth, and N utilization. Increases in serum ALB and total cholesterol are indicative of the positive effects of CaPr supplementation on retained N and lipogenesis. Although the calves received the same diet (Table 1) and had a similar DMI, fat deposition was greater in calves that received CaPr supplementation.

It has been proposed that supplemental CaPr may increase blood Ca levels. But this effect has only been observed with high levels of supplemental Ca [49]. The Ca provided from CaPr in the current study was only 4.2 g. Increases in serum AST, ALT, and NEFA have been observed with CaPr [50], but at levels of supplementation 6.5-fold that of the present study. Increased serum CRE has been observed with CaPr ingestion, but at levels 4-fold that of the present study [13].

### 4.4. Cost and Income Economics Analysis

Using the profit estimated for the control group as a baseline, bull calves that received CaPr supplementation for 14, 28, and 56 d had a very similar profit (~USD 6.80/calf). Because the cost of gain was very similar for these treatments and the controls (USD 1.42 vs. 1.46/kg), the positive difference in profit was mediated mainly by an increase in income selling (+USD 13.02/calf) for the CaPr calves. For the calves that received CaPr for 42 d, compared to the controls, the profit controls were greater than +USD 34.84/calf, both due to an increase in income selling (+USD 41.99/calf) and a reduction in the cost of gain (USD 1.26 vs. 1.46/kg BW).

It is important to highlight that this study is limited in its number of animals, an increase in which would allow for the extrapolation of the economic results to a larger scale. In this sense, in our experiment, there was no mortality or morbidity during this stage, a situation that does not occur in the fattening system.

## 5. Conclusions

Based on the performance, serum metabolites, and profit, the optimal duration of supplemental CaPr was 42 d. Lesser or greater periods of CaPr supplementation did not appreciably enhance the calf growth performance when evaluated following 56 d on feed. Offering feedlot calves 19 g/d CaPr during the initial 42 d period following arrival enhanced the growth performance and efficiency of the dietary energy utilization, resulting in greater economic returns.

It is important to note that this is a pioneering study on the topic, contributing to our understanding of the strategy of using glucogenic compounds as an alternative to improving the productivity and health during the receiving phase of cattle. Therefore, further studies addressing other physiological and metabolic variables involved are needed to better understand the mechanisms by which CaPr provides benefits to cattle during this stage. Likewise, larger-scale trials are needed to assess the extrapolation potential of the experimental results to feedlot systems.

## Figures and Tables

**Table 1:** Ingredients of the basal diet and nutritional composition (dry matter basis) offered to high-risk bull calves.

Ingredients	% of Dietary DM
Alfalfa hay mature	25.0
Oats hay	25.0
Coarse-ground corn grain	28.0
Soybean meal-44	10.5
Molasses cane	5.0
Vegetable fat (soybean oil)	2.15
Sodium bentonite	0.75
Sodium sesquicarbonate	1.5
Calcium carbonate	0.8
Monocalcium phosphate	0.2
Urea	0.5
Salt	0.5
Premix ^a^	0.1
Chemical composition %	
Dry matter (DM)	87.24
Crude protein (CP)	14.88
Ether extract (EE)	4.36
Neutral detergent fiber (NDF)	34.84
Calcium ^b^	0.93
Phosphorus ^b^	0.29
Ca/P ratio	0.32
Calculated net energy, Mcal/kg ^b^	
Maintenance	1.57
Gain	0.98

^a^ Contained the following per kilogram of premix: 0.5 g of Co, 50 g of Fe, 2.5 g of I, 50 g of Mn, 50 g of Zn, 0.2 g of Se, and 15 g of Cu, as well as 5,000,000 IU of vitamin A, 2,000,000 IU of vitamin D, and 10,000 IU of vitamin E. ^b^ Calculated based on the tabular values for individual feed ingredients (Ca, P, NE_m_, and NE_g_) [18], with the exception of DM, CP, EE [19], and NDF (Ankom procedures, Macedon, NY, USA), which were determined in our laboratory.

**Table 2:** Growth performance of high-risk bull calves supplemented with calcium propionate (CaPr) for periods of 14, 28, 42, or 56 days after arrival to the feedlot.

**Item ^a^**	**Days on CaPr After Arrival ^b^**									**SEM ^c^**
	**Control**	**14**		**28**		**42**		**56**		
IBW, kg	153.17	157		159.62		154.12		157.87		3.721
FBW, kg	221.33 ^b^	228.25 ^ab^		231.63 ^ab^		233.88 ^a^		229.88 ^ab^		4.23
ADG, kg/d	1.217 ^b^	1.272 ^b^		1.286 ^b^		1.424 ^a^		1.286 ^b^		0.044
DMI, kg/d	4.888	4.964		4.958		4.966		4.898		0.043
ADG/DMI ratio	0.249 ^bc^	0.256 ^b^		0.259 ^b^		0.287 ^a^		0.262 ^b^		0.008
**Contrast**	***p*-Value**									
	**0 vs. 14**	**0 vs. 28**	**0 vs. 42**	**0 vs. 56**	**14 vs. 28**	**14 vs. 42**	**14 vs. 56**	**28 vs. 42**	**28 vs. 56**	**42 vs. 56**
IBW	0.44	0.20	0.85	0.35	0.57	0.53	0.85	0.24	0.70	0.42
FBW	0.35	0.23	<0.01	0.25	0.75	0.03	0.80	0.06	0.94	0.05
ADG	0.42	0.32	<0.01	0.32	0.83	0.02	0.83	0.03	1.00	0.03
DMI	0.25	0.29	0.24	0.89	0.93	0.97	0.28	0.90	0.32	0.27
ADG/DMI	0.58	0.44	0.01	0.32	0.81	0.02	0.64	0.03	0.81	0.05

^a^ IBW = initial body weight, FBW = final body weight, ADG = average daily gain, and DMI = dry matter intake. ^b^ Treatments consisting of the oral administration of CaPr at a dose of 19 g/calf/d (Propical^®^, Dresen Química, SAPI de CV., Ciudad de México, México) at four supplementation periods of 14, 28, 42, or 56 d after the high-risk calves arrived at the feedlot. ^c^ SEM = standard error of the mean. ^a,b,c^ Rows with different superscripts differ (*p* ≤ 0.05) according to Tukey’s test.

**Table 3:** Dietary energetics and ultrasound measurements of high-risk bull calves supplemented with calcium propionate (CaPr) at periods of 14, 28, 42, or 56 days after arrival to the feedlot.

**Item ^a^**	**Days on CaPr After Arrival ^b^**									**SEM ^c^**
	**Control**	**14**		**28**		**42**		**56**		
Dietary net energy (Mcal/kg)										
Maintenance	1.883 ^b^	1.936 ^ab^		1.969 ^ab^		2.072 ^a^		1.979 ^ab^		0.032
Gain	1.249 ^b^	1.288 ^ab^		1.317 ^ab^		1.407 ^a^		1.326 ^ab^		0.027
Observed-to-expected dietary NE										
Maintenance	1.204 ^b^	1.233 ^ab^		1.254 ^ab^		1.320 ^a^		1.261 ^ab^		0.026
Gain	1.274 ^b^	1.314 ^ab^		1.344 ^ab^		1.436 ^a^		1.353 ^ab^		0.037
Ultrasound measurement										
FAT (mm)	2.848	3.391		3.004		3.399		3.439		0.222
RFT (mm)	4.080 ^bc^	3.825 ^c^		3.733 ^c^		4.467 ^ab^		4.935 ^a^		0.231
LMA (cm^2^)	35.756	36.664		35.706		35.57		36.321		0.704
**Contrast**	***p*-Value**									
	**0 vs. 14**	**0 vs. 28**	**0 vs. 42**	**0 vs. 56**	**14 vs. 28**	**14 vs. 42**	**14 vs. 56**	**28 vs. 42**	**28 vs. 56**	**42 vs. 56**
Dietary net energy (Mcal/kg)										
Maintenance	0.42	0.19	<0.01	0.14	0.58	0.01	0.47	0.03	0.86	0.04
Gain	0.42	0.19	<0.01	0.14	0.58	0.01	0.47	0.03	0.86	0.04
Observed-to-expected dietary NE										
Maintenance	0.42	0.19	<0.01	0.14	0.58	0.01	0.47	0.03	0.86	0.04
Gain	0.42	0.19	<0.01	0.14	0.58	0.01	0.47	0.03	0.86	0.04
Ultrasound measurement										
FAT (mm)	0.09	0.61	0.11	0.09	0.17	1.00	0.90	0.21	0.17	0.91
RFT (mm)	0.45	0.34	0.25	0.03	0.77	0.04	<0.01	0.03	<0.01	0.20
LMA (cm^2^)	0.41	0.99	0.11	0.90	0.39	0.12	0.45	0.84	0.91	0.17

^a^ FAT = 12th rib fat thickness, RFT = rump fat thickness, and LMA = *Longissimus lumborum* muscle area. ^b^ Treatments consisting of the oral administration of CaPr at a dose of 19 g/calf/d (Propical^®^, Dresen Química, SAPI de CV., Ciudad de México, México) at four supplementation periods of 14, 28, 42, or 56 d after the high-risk calves arrived at the feedlot. ^c^ SEM = standard error of the mean. ^a,b,c^ Rows with different superscripts differ (*p* ≤ 0.05) according to Tukey’s test.

**Table 4:** Enzymic activity of high-risk bull calves supplemented with calcium propionate (CaPr) at periods of 14, 28, 42, or 56 d after arrival to the feedlot.

**Item ^a^**	**Days on CaPr After Arrival ^b^**					**SEM ^c^**		**Reference Intervals ^d^**		
	**Control**	**14**	**28**	**42**	**56**					
ALP, U/I	242.77	211.5	190.7	246	233.1	34.694		14.4–469.6 (209.7 ± 107)		
GGT, U/I	17.05	13.36	16.25	19.6	16.46	3.779		10.0–47.9 (17.0 ± 8.9)		
AST, U/I	80.64	83.44	72.61	83.1	74.69	10.025		38.9–124.2 (68.8 ± 21.9)		
ALT, U/I	29.63	29.99	28.22	31.7	33.46	2.021		16.6–44.4 (26.5 ± 7.3)		
**Contrast**	***p*-Value**									
	**0 vs. 14**	**0 vs. 28**	**0 vs. 42**	**0 vs. 56**	**14 vs. 28**	**14 vs. 42**	**14 vs. 56**	**28 vs. 42**	**28 vs. 56**	**42 vs. 56**
ALP	0.57	0.06	0.96	0.85	0.17	0.46	0.55	0.05	0.08	0.80
CGT	0.27	0.46	0.95	0.48	0.06	0.30	0.08	0.42	1.00	0.45
AST	0.85	0.58	0.87	0.71	0.48	0.98	0.57	0.51	0.90	0.62
ALT	0.90	0.63	0.48	0.22	0.58	0.55	0.29	0.28	0.09	0.60

^a^ ALP = alkaline phosphatase, GGT = gamma glutamyltransferase, AST = aspartate aminotransferase, and ALT = alanine aminotransferase. ^b^ Treatments consisting of the oral administration of CaPr at a dose of 19 g/calf/d (Propical^®^, Dresen Química, SAPI de CV., Ciudad de México, México) at four supplementation periods of 14, 28, 42, or 56 d after the high-risk calves arrived at the feedlot. ^c^ SEM = standard error of the mean. ^d^ Reference intervals reported here are from the publication by Carrillo-Muro et al. [17]. Rows with different superscripts differ (*p* ≤ 0.05) according to Tukey’s test.

**Table 5:** Serum metabolites of high-risk bull calves supplemented with calcium propionate (CaPr) at periods of 14, 28, 42, or 56 d after arrival to the feedlot.

**Item ^a^**	**Days on CaPr After Arrival ^b^**					**SEM ^c^**		**Reference Intervals ^d^**		
	**Control**	**14**	**28**	**42**	**56**					
TP, g/dL	6.95	6.96	7.10	6.88	7.01	0.307		4.4–7.71 (6.22 ± 0.83)		
ALB, g/dL	3.06 ^b^	3.10 ^b^	3.34 ^ab^	3.20 ^ab^	3.56 ^a^	0.096		1.9–3.7 (2.97 ± 0.50)		
GLO, g/dL	3.94	3.86	3.66	3.76	3.40	0.237		2.2–4.11 (3.18 ± 0.50)		
ALB/GLO ratio	0.77	0.84	0.94	0.85	1.05	0.065		0.68–1.32 (0.94 ± 0.17)		
BUN, mg/dL	13.24	12.67	12.06	12.17	10.03	0.942		6.91–16.1 (11.08 ± 2.31)		
CRE, mg/dL	0.80	0.73	0.74	0.77	0.85	0.050		0.52–1.35 (0.81 ± 0.20)		
TBIL, mg/dL	0.42	0.16	0.44	0.34	0.24	0.158		0.20–1.30 (0.34 ± 0.29)		
TCHO, mg/dL	81.41 ^b^	98.93 ^ab^	112.36 ^a^	113.04 ^a^	105.60 ^ab^	8.612		50.0–127.7 (78.6 ± 22.0)		
TG, mg/dL	20.39	31.33	22.70	28.89	28.79	8.524		10.0–360.7 (36.5 ± 77.1)		
Ca, mg/dL	11.32	11.83	11.05	11.04	10.63	0.314		7.12–12.5 (10.28 ± 1.42)		
GLU, mg/dL	94.65	105.93	100.84	102.40	87.70	8.826		26.1–126.0 (89.0 ± 22.5)		
**Contrast**	***p*-Value**									
	**0 vs. 14**	**0 vs. 28**	**0 vs. 42**	**0 vs. 56**	**14 vs. 28**	**14 vs. 42**	**14 vs. 56**	**28 vs. 42**	**28 vs. 56**	**42 vs. 56**
TP	0.61	0.73	0.79	0.83	0.86	0.86	0.79	0.98	0.91	0.94
ALB	0.33	0.05	0.12	0.01	0.30	0.52	0.02	0.80	<0.01	0.01
GLO	0.79	0.45	0.63	0.49	0.59	0.78	0.37	0.84	0.18	0.29
ALB/GLO	0.64	0.51	0.97	0.11	0.27	0.64	0.22	0.56	0.03	0.12
BUN	0.70	0.61	0.07	0.67	0.92	0.11	0.40	0.13	0.36	0.07
CRE	0.33	0.48	0.72	0.53	0.83	0.58	0.13	0.74	0.20	0.37
TBIL	0.21	0.91	0.73	0.42	0.19	0.42	0.71	0.65	0.36	0.65
TCHO	0.11	0.03	0.03	0.10	0.25	0.22	0.32	0.95	0.77	0.63
TG	0.76	0.18	0.93	0.60	0.26	0.86	0.73	0.28	0.56	0.68
Ca	0.28	0.56	0.58	0.18	0.12	0.12	0.09	0.97	0.39	0.47
GLU	0.19	0.28	0.23	0.26	0.37	0.56	0.09	0.80	0.14	0.14

^a^ TP = total protein, ALB = albumin, GLO = globulin, BUN = blood urea nitrogen, CRE = creatinine, TBIL = total bilirubin, TCHO = total cholesterol, TG = triglyceride, Ca = calcium, and GLU = glucose. ^b^ Treatments consisting of the oral administration of CaPr at a dose of 19 g/calf/d (Propical^®^, Dresen Química, SAPI de CV., Ciudad de México, México) at four supplementation periods of 14, 28, 42, or 56 d after the high-risk calves arrived at the feedlot. ^c^ SEM = standard error of the mean. ^d^ Reference intervals reported here are from the publication by Carrillo-Muro et al. [17]. ^a,b^ Rows with different superscripts differ (*p* ≤ 0.05) according to Tukey’s test.

**Table 6:** Electrolytes of high-risk bull calves supplemented with calcium propionate (CaPr) at periods of 14, 28, 42, or 56 d after arrival to the feedlot.

**Item ^a^**	**Days on CaPr After Arrival ^b^**					**SEM ^c^**		**Reference Intervals ^d^**		
	**Control**	**14**	**28**	**42**	**56**					
Na^+^, mEq/L	127.34	131.82	124.64	126.91	124.01	3.066		98.2–143.0 (126.3 ± 12.1)		
K^+^, mEq/L	4.39	4.17	4.04	3.85	4.05	0.261		3.11–8.59 (4.93 ± 1.21)		
Cl^−^, mEq/L	91.95	94.89	88.98	90.06	86.18	2.699		71.1–109.0 (90.8 ± 9.8)		
**Contrast**	***p*-Value**									
	**0 vs. 14**	**0 vs. 28**	**0 vs. 42**	**0 vs. 56**	**14 vs. 28**	**14 vs. 42**	**14 vs. 56**	**28 vs. 42**	**28 vs. 56**	**42 vs. 56**
Na^+^	0.32	0.54	0.93	0.48	0.13	0.31	0.12	0.64	0.89	0.57
K^+^	0.54	0.33	0.19	0.40	0.73	0.45	0.78	0.63	0.98	0.63
Cl^−^	0.38	0.38	0.60	0.13	0.10	0.19	0.03	0.77	0.44	0.33

^a^ Na^+^ = sodium, K^+^ = potassium, and Cl^−^ = chlorine. ^b^ Treatments consisting of the oral administration of CaPr at a dose of 19 g/calf/d (Propical^®^, Dresen Química, SAPI de CV., Ciudad de México, México) at four supplementation periods of 14, 28, 42, or 56 d after the high-risk calves arrived at the feedlot. ^c^ SEM = standard error of the mean. ^d^ Reference intervals reported here are from the publication by Carrillo-Muro et al. [17].

**Table 7:** Cost and income economics estimated for high-risk bull calves supplemented with calcium propionate (CaPr) at periods of 14, 28, 42, or 56 d after arrival to the feedlot.

Item	Control	Days on CaPr After Calves Arrive ^a^			
		14	28	42	56
Days on feed	56	56	56	56	56
Days on CaPr supplementation	0	14	28	42	56
No. of calves	10	10	10	10	10
Growth performance					
Initial, kg	153.17	157.00	159.62	154.12	157.87
Final, kg	221.33	228.25	231.63	233.88	229.88
Dry matter intake, kg/day	4.89	4.96	4.96	4.97	4.90
Processing practice costs, USD/calf					
Preventative health	USD 0.43	USD 0.43	USD 0.43	USD 0.43	USD 0.43
Deworming	USD 0.75	USD 0.75	USD 0.75	USD 0.75	USD 0.75
Pour-on	USD 0.65	USD 0.65	USD 0.65	USD 0.65	USD 0.65
Metaphylatic antimicrobial treatment	USD 1.38	USD 1.38	USD 1.38	USD 1.38	USD 1.38
Ear tags	USD 0.61	USD 0.61	USD 0.61	USD 0.61	USD 0.61
Subtotal	USD 3.82	USD 3.82	USD 3.82	USD 3.82	USD 3.82
Feed costs, USD/calf					
Feed ^b^	USD 95.57	USD 96.94	USD 96.94	USD 97.13	USD 95.77
CaPr supplementation ^c^	USD 0.00	USD 0.59	USD 1.18	USD 1.76	USD 2.35
Subtotal	USD 95.57	USD 97.53	USD 98.11	USD 98.90	USD 98.12
Total cost ^d^	USD 99.39	USD 101.35	USD 101.93	USD 102.72	USD 101.94
Income, USD/calf					
Income (selling calves) ^e^	USD 246.74	USD 257.92	USD 260.68	USD 288.73	USD 260.67
Net income ^f^	USD 147.34	USD 156.57	USD 158.74	USD 186.01	USD 158.73
Difference ^g^	-	USD 5.41 *	USD 7.58 *	USD 34.84 ***	USD 7.57 *
Cost of gain, USD/kg ^h^	USD 1.46	USD 1.42	USD 1.42	USD 1.29 **	USD 1.42

^a^ Treatments consisting of the oral administration of CaPr at a dose of 19 g/calf/d (Propical^®^, Dresen Química, SAPI de CV., Ciudad de México, México) at four supplementation periods of 14, 28, 42, or 56 d after the high-risk calves arrived at the feedlot. ^b^ Feed cost = (DMI, kg/d × price of feed kg) × days on feed, where the price of feed kg is USD 0.349 × 56 d. ^c^ CaPr supplementation = (CaPr, kg/d × price of CaPr kg) × days on supplementation, where the price of CaPr kg is USD 2.1 × 14, 28, 42, or 56 d supplementation. ^d^ Cost total = processing practice + feed + CaPr supplementation. ^e^ Income (selling calves) = (weight out − weight in) × price of BW/kg to calves, where the selling price by BW is USD 3.62. ^f^ Net income = income (selling calves) − total cost. ^g^ Difference = CaPr treatments–control. ^h^ Cost of gain = total cost/(FBW − IBW). * *p* < 0.05, ** *p* < 0.01, and *** *p* < 0.001 without CaPr versus supplemented with CaPr.

## Data Availability

The information published in this study is available on request from the corresponding author.
